# Clinical Outcomes in Neurologically Intact Children With Small Intracranial Bleeds and Simple Skull Fractures

**DOI:** 10.7759/cureus.42848

**Published:** 2023-08-02

**Authors:** Mohamed Almuqamam, Tina C Loven, Lindsay G Arthur III, Norrell K Atkinson, Harsh Grewal

**Affiliations:** 1 Pediatric Critical Care Medicine, St. Christopher’s Hospital for Children, Drexel University College of Medicine, Philadelphia, USA; 2 Neurosurgery, St. Christopher’s Hospital for Children, Drexel University College of Medicine, Philadelphia, USA; 3 Pediatric Surgery, St. Christopher’s Hospital for Children, Drexel University College of Medicine, Philadelphia, USA; 4 Child Protection Program, St. Christopher’s Hospital for Children, Drexel University College of Medicine, Philadelphia, USA

**Keywords:** pediatric head trauma, mild traumatic brain injury, traumatic intracranial hematoma, skull fractures, closed head injury

## Abstract

Introduction

Children with minor intracranial hemorrhage (ICH) and/or simple skull fractures are often hospitalized for monitoring; however, the majority do not require any medical, surgical, or critical care interventions. Our purpose was to determine the rate of significant clinical sequela (SCS) and identify associated risk factors in neurologically intact children with close head trauma.

Methods

This is a retrospective observational study. Children (≤ 3 years of age) admitted with closed head trauma, documented head injuries (ICH ≤ 5mm and/or simple skull fracture), and a Glasgow Coma Scale (GCS) score of ≥14, between January 2015 and January 2020, were included. We collected demographics, resource utilization, and patient outcomes variables. SCS was defined as any radiologic progression, and/or clinically important medical or neurological deterioration.

Results

A total of 205 patients were enrolled in the study (65.4% male, mean age 7.7 months). Repeat neuroimaging was obtained in 41/205 patients (20%) with radiologic progression noted in 5/205 (2.4%). Thirteen out of 205 patients (6.3%) experienced SCS. Patients with SCS were more likely to be males (92.3% vs 63.5% in females, P=0.035) to have had a report filed with child protective services due to a concern for abuse/neglect (92.3% vs 61.5% in females, P=0.025), and to have had a non-linear skull fracture (P<0.001). No other factors were shown to be predictive of SCS with enough statistical significance.

Conclusion

Neurologically intact children with traumatic closed head injury are at low risk for developing SCS. This study suggests that most of these children may not need ICU monitoring. This study also showed that a certain subset might be at an increased risk of developing SCS.

## Introduction

Traumatic brain injury (TBI) is a leading cause of death and disability worldwide, especially in young children. Intracranial hemorrhage (ICH) in children, while less common than in adults, is a serious and life-threatening diagnosis [1]. Children are also more susceptible to traumatic skull fractures given the thin and more pliable nature of their skulls. Multiple studies have documented different incidence rates of ICH and skull fractures among children, but the exact incidence rate, prevalence, and treatment are not fully known [2-4]. Various factors influence the management approach of ICH and skull fractures including etiology, location, size, presence of mass effect, hemodynamic stability, and the neurosurgeon’s preference [5].

There are no validated prognostic tools for predicting clinical course and outcomes in children with TBI with the majority of available data being derived from a small number of cohort studies [6]. On the other hand, TBI in adults is well-studied with multiple validated tools including the ICH score which was developed more than 10 years ago and has been helping physicians predict outcomes for adults suffering from ICH [7,8]. However, tools developed and validated in adults may be difficult to apply in children because causes, comorbidities, and survival rates in children with TBI may differ from that of adults [9].

Patients with mild TBI and visible intracranial injuries on imaging are a distinct group of patients, the management of whom is controversial. Current international guidelines recommend routine hospital admission for all patients with mild TBI who have documented injuries [10]. Nonetheless, only a small proportion of these patients seem to require neurosurgical or critical care interventions [10]. The same uncertainty applies to children with mild TBI, the management of whom varies significantly among and within institutions. The presence or absence of ICH on the initial imaging tends to dominate the clinical decision, often committing the child to an observation unit to monitor for potential complications.

Few studies evaluated the clinical course and outcomes of pediatric patients with mild TBI, isolated skull fracture (ISF), and small ICH. Rollins et al. evaluated ISF and found that asymptomatic children who present with a Glasgow Coma Scale (GCS) of 15 and a nondisplaced or minimally displaced ISF without other intracranial injuries can be considered for discharge from the emergency room after a short period of observation [11]. Another study by Arrey et al. came to the same conclusion but advised that patients with mental status changes, additional intracranial injuries, or possible nonaccidental injury may require observation [12]. Holsti et al. examined clinical outcomes in pediatric patients with closed head injuries who were admitted to an observation unit in the emergency department and found that 96% of patients were discharged safely within 24 hours without serious complications [13]. Greenberg et al. evaluated pediatric patients with mild TBI and ICH and found that only 6.8% experienced neurological decline and 5.1% required neurosurgical intervention [14].

We hypothesized that neurologically intact children with minor ICH and/or simple skull fracture have a low rate of adverse outcomes during the hospital course and that certain patient-specific and radiological factors can be predictive of the risk of clinical deterioration. The purpose of this study was to determine the rate of significant clinical sequela (SCS) in this patient population and to determine associated factors to help identify patients that need closer monitoring.

This article was previously presented as a meeting abstract at the American Pediatric Surgical Association (APSA) annual meeting on May 20, 2021.

## Materials and methods

This is a retrospective chart review study of children presenting to a level one trauma center, St. Christopher's Hospital for Children. Philadelphia, Pennsylvania, United States, during a five-year period (January 2015 to January 2020). The Institutional Review Board at Drexel University (IRB # 2003007715) approved all research procedures and waived the need for signed informed consent. Inclusion criteria were children (≤ 3 years of age) presenting with closed head trauma and documented intracranial injuries on initial neuroimaging (minor ICH and/or simple skull fracture) and an initial GCS of ≥14. We excluded the following patients: those with severe multiple injuries unrelated to TBI that would necessitate an ICU stay otherwise (e.g. pneumothorax, visceral hemorrhage), patients with penetrating intracranial injuries (e.g. stab, gunshot wound, and animal bites), patients requiring mechanical ventilation, patients with pre-existing cardiovascular or oncological disease, patients with recent neurosurgeries or transfusions, and patients with incomplete medical records.

We collected demographics, resource utilization, and patient outcomes variables. Age, gender, mechanism of injury, GCS score, and non-accidental trauma (NAT) report filing with child protective services were collected for demographics. Emergency department length of stay (LOS), hospital LOS, level of trauma activation, rate of reimaging, transfer between floor and ICU, return to ED within 60 days, and re-admission within 60 days were collected for resource utilization. Type of ICH, size of ICH, presence of midline shift, presence of skull fracture, type of skull fracture, displacement of skull fracture, GCS deterioration, medical or surgical interventions, and rate of SCS were collected for patient outcomes.

Minor ICH was defined as any subarachnoid hemorrhage (SAH), epidural hemorrhage (EDH), or subdural hemorrhage (SDH) measuring 5mm or less on the initial brain scan. Simple skull fracture was defined as any closed and none-to-minimally displaced fracture of the skull. SCS definition was developed from the work done by Greenberg et al. in 2014, Washington et al. in 2012, and the Pediatric Emergency Care Applied Research Network (PECARN) group in 2009 [14-16]. SCS was defined as any radiologic progression of hemorrhage within seven days of injury (evident by an increase in ICH volume >30% or a new ICH), any medical deterioration (defined as worsening cardiopulmonary status, new electrolyte disturbances, or new events necessitating ICU monitoring), and any clinically important neurological deterioration noted within seven days of injury (defined as death from TBI, neurological decline including new altered mental status or status epilepticus, and neurosurgical intervention including intracranial pressure monitoring, ventriculostomy, hematoma evacuation, lobectomy, tissue debridement, and dura repair). Each SCS represents a unique patient.

Statistical analysis was performed using IBM SPSS Statistics for Windows, Version 25.0 (Released 2017; IBM Corp., Armonk, New York, United States). The data reported included both demographic variables as well as study variables that were discrete and continuous. Discrete variables were reported as count and percent within categories, and continuous variables were reported as mean and standard deviation. Comparisons between groups were made with Fisher's exact and Chi-square tests of association. As an exploratory study, there were no corrections applied to the data (p-values), and an omnibus p-value of 0.05 was used to determine statistical significance. There were no imputation methods applied to the data for missing values. Variables with missing values were reported for observed data only.

## Results

**Figure 1:**
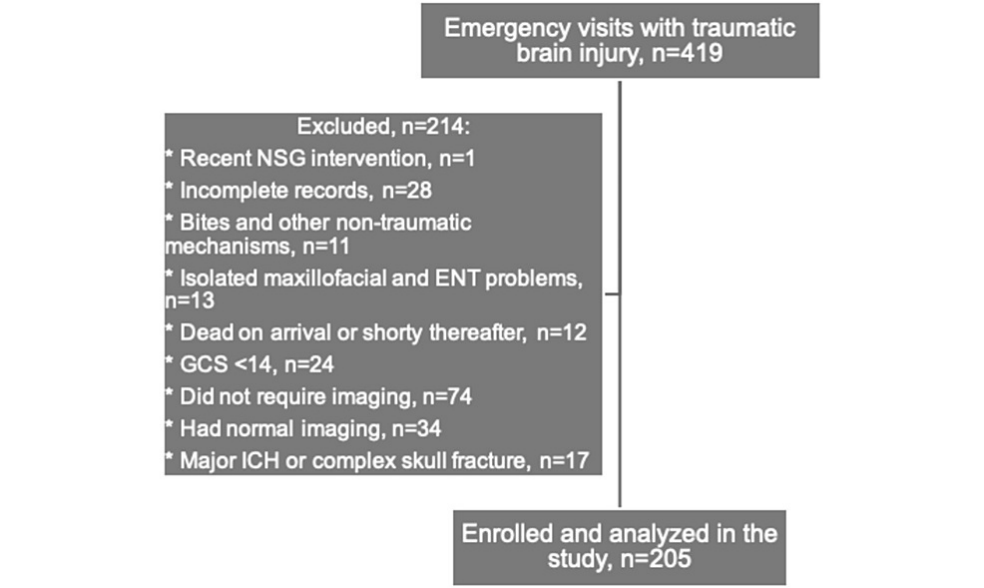
Study CONSORT diagram GCS: Glasgow Coma Scale; ICH: intracranial hemorrhage; CONSORT: Consolidated Standards of Reporting Trials; NSG: neurosurgical

There were 419 patients with closed head trauma identified within the local trauma registry database (Figure 1). The following patients were excluded: one patient due to a recent neurosurgical intervention, 28 patients had incomplete records, 11 patients presented with non-traumatic mechanisms, 13 patients had isolated maxillofacial and ENT problems, 12 patients were dead on arrival or shortly thereafter, 24 patients had GCS < 14, 74 patients did not undergo brain imaging, 34 patients had normal brain imaging, and 17 patients had either major ICH (> 5mm) or complex skull fracture. A total of 205 patients were therefore included in the study, out of which 21 patients had isolated minor ICH, 111 patients had isolated simple skull fracture, and 73 patients had a combination of minor ICH and simple skull fracture.

Table 1 demonstrates baseline patient characteristics. The mean age of the study population was 7.7 months (SD 6.63), two-thirds of whom were males (65.4%). Eighy-five percent of our cohort were triaged as a trauma level three, 13% were a level two, and 2% were a level one. The most common (65%) mechanism of injury was fall. Filing with child protective services for NAT concerns occurred in 63% of cases. At presentation, 163 patients were asymptomatic (80%) whereas 42 patients presented with at least one symptom, somnolence being the most common.

**Table 1:** Demographics of study population MVC: motor vehicle collision; NAT: non-accidental trauma; LOC: loss of consciousness

Characteristics	Value
Age (in months), mean (SD)	7 (6.63)
Gender, N (%)	
Male	134 (65.4)
Female	71 (34.6)
Trauma activation level, N (%)	
Level I	4 (1.9)
Level II	27 (13.3)
Level III	172 (84.7)
Mechanism of injury, N (%)	
MVC	8 (3.9)
Fall	134 (65.4)
NAT	63 (30.7)
NAT filing, N (%)	
Yes	130 (63.4)
No	75 (36.6)
Symptoms on presentation, N (%)	
No	163 (79.5)
Yes	42 (20.5)
Seizure	1 (0.5)
LOC	5 (2.5)
Vomiting	7 (3.4)
Somnolence	15 (7.3)
Fussy	9 (4.4)
Fever	2 (1.0)
Other	2 (1.0)

Details of the neuroimaging findings of the study population are shown in Table 2. Of the 205 patients, 94 were found to have an ICH on initial imaging. The most common type of ICH in our patient cohort was SDH (54.2%) followed by SAH (19.1%) and a combination of both types (11.7%). Around half of ICH cases were trace, 1mm in size (47.8%) followed by 5mm (19.1%), 3mm (12.7%), 4mm (10.6%), and 2mm (9.5%). Five out of the 205 patients (2.5%) were found to have midline shift and 184 patients (89.7%) had a skull fracture. Repeat neuroimaging was obtained at least once in 41/205 patients (20%), two patients were reimaged twice, and one patient received reimaging more than twice. Of the 41 patients who received reimaging, six patients developed new symptoms and/or new abnormal exam findings (one had a brief episode of altered mental status, three patients developed status epilepticus, one patient had a new focal neurological deficit, and one developed syndrome of inappropriate antidiuretic hormone secretion (SIADH)). The rest of the repeat imaging cases were either routine reimaging as advised by neurosurgery or as part of the evaluation for NAT. The isolated ICH group (33%) and combined (ICH and skull fracture) group (36%) were more likely to receive repeat imaging than the ISF group (4%; p <0.05). Radiologic progression was noted in 5/41 (12.2% of repeat studies and 2.4% of the study population). Only two out of five with radiologic progression had new symptoms and/or abnormal findings that prompted the repeat imaging.

**Table 2:** Radiologic characteristics of study population ICH: intracranial hemorrhage; SAH: subarachnoid hemorrhage; SDH: subdural hemorrhage; EDH: epidural hemorrhage

Radiological Findings	N (%)
Type of ICH	
SDH	51 (54.2)
SDH and SAH	11 (11.7)
EDH	8 (8.5)
SAH	18 (19.0)
Intracerebral hemorrhage	5 (5.3)
Intracerebral hemorhage and SAH	1 (1.0)
Size	
1mm (trace)	45 (47.8)
2mm	9 (9.5)
3mm	12 (12.7)
4mm	10 (10.6)
5mm	18 (19.1)
Midline shift	
Yes	5 (2.5)
No	200 (97.5)
Skull fracture	
Yes	184 (89.7)
No	21 (10.2)
Number of repeat head imaging	
Once	38 (18.5)
Twice	2 (1.5)
>2	1 (0.5)
Result of repeat head imaging	
Same findings	32 (78.0)
New ICH	4 (9.7)
Worsening ICH	1 (2.4)
Disappearance of ICH	4 (9.7)

Description of the hospital course and clinical outcomes of the study population are shown in Table 3. The average emergency department LOS for all patients was 215 minutes (SD 19) and the average hospital stay was 44 hours (SD 7). No mortalities were reported in our sample during their hospital stay. Among the 205 patients, 13 patients (6.3%) experienced SCS (four patients developed new ICH, one patient developed progression of initial ICH, four patients developed status epilepticus, three patients underwent ICH evacuation, and one patient developed clinically significant SIADH). The median time to SCS development was 23 hours (IQR 30-12).

**Table 3:** Clinical outcomes of study population AMS: altered mental status; SIADH: syndrome of inappropriate antidiuretic hormone secretion; ENT: ear, nose, and throat; EVD: external ventricular drain; ED: emergency department; VP: ventriculoperitoneal; SSI: surgical site infection

Outcomes	N (%)
Acute neurological problem	
AMS	3 (1.4)
Seizure	3 (1.4)
Loss of pupillary reflex	1 (0.5)
Head swelling	1 (0.5)
Acute medical problem	
Respiratory distress	3 (1.4)
SIADH	1 (0.5)
Acute surgical problem	
Unrecognized bone fracture	2 (1.0)
ENT procedure	2 (1.0)
Hematoma Evacuation	3 (1.5)
EVD	1 (0.4)
Return to ED within 60 days	39 (19.0)
Reason for readmission within 60 days	
Due to worsening head swelling	2 (0.9)
Due to trauma to head	3 (1.4)
Due to VP shunt malfunction	2 (0.9)
Due to weakness	1 (0.5)
Due to SSI	1 (0.5)
Due to unrelated medical/surgical complaints	4 (1.9)

Patients with SCS were more likely to be males (92.3% vs 63.5% females, P=0.035). Patients with SCS were also more likely to have had a NAT report filed with child protective services (92.3% vs 61.5%, P=0.025) (Table 4). On further analysis of children with NAT filing, evaluation by the hospital’s child abuse team concluded that 75% of those children who developed SCS had injuries that were most consistent with abuse/non-accidental trauma, whereas only 18.8% were in patients without SCS (P<0.001). As Table 4 shows, no other patient-specific factors were shown to be predictive of SCS with enough statistical significance. SCS was more common in symptomatic patients, one of the five patients presenting with seizure developed SCS (20%) versus 12 out of 200 patients without seizures (6%) (P=0.2). Three out of 42 symptomatic patients (other than seizure) developed SCS (7.1%) versus 10 out of 163 (6.1%) (P=0.8). The rate of SCS among patients with ICH (8.8%) was twice that of patients with ISF (4.3%) (P=0.18). Eight out of the 13 patients with SCS had ICH on the initial scan (SDH=7 and EDH=1). Around half of the SCS group had a combination injury (ICH and skull fracture) versus ISF or isolated ICH (46.2% vs 38.5% vs 15.4%, P=0.41). Unsurprisingly, the size of ICH also seems to correlate with the risk of SCS; patients with SCS were more likely to have an ICH of ≥3mm on their initial imaging (62.5% vs 41.5%, P=0.57).

**Table 4:** Significant clinical sequelae and associated patient factors MVC: motor vehicle collision; NAT: non-accidental trauma ^a^ P-values reported from Chi-square test^; b^ P-values reported from Fisher’s exact test

Patient Factors	N (%)	P-values
Gender		0.035^a^
Male	12 (92.3)	
Female	1 (7.7)	
Mechanism of injury		0.099^b^
MVC	0 (0.0)	
Fall	5 (38.5)	
Unexplained	8 (61.5)	
NAT filing		0.025^ a^
Yes	12 (92.3)	
No	1 (7.7)	
NAT filing outcome		<0.001^ a^
Abuse	9 (75)	
Accidental injury	2 (16.7)	
Indeterminate	1 (8.3)	
Seizure on presentation		0.205^ a^
Yes	1 (7.7)	
No	12 (92.3)	
Symptomatic on presentation (other than seizure)		0.811^ a^
Yes	3 (23.1)	
No	10 (76.9)	

As Table 5 shows, patients with SCS were less likely to have a midline shift or skull fracture than non-SCS (0% vs 2.6%, P=0.55 and 84.6% vs 90.1%, P=0.52, respectively). The most common location of skull fracture among both SCS and non-SCS groups was parietal (72.7% vs 74%, P=0.56) while occipital occurred in 18.2% of SCS group versus 9.8% in non-SCS group. Comminuted, depressed, and combined types of fractures were more common among patients with SCS than patients without (18.2%, 18.2%, and 18.2% vs 4.6%, 8.1%, and 0.6%, P <0.001).

**Table 5:** Significant clinical sequelae and associated radiological factors ICH: intracranial hemorrhage; SAH: subarachnoid hemorrhage; SDH: subdural hemorrhage; EDH: epidural hemorrhage ^a^ P-value reported from Chi-square test; ^b^ P-values reported from Fisher’s exact test

Radiological Factors	N (%)	P-values
ICH presence		0.186^ a^
Yes	8 (61.5)	
No	5 (38.5)	
Type of ICH		0.39^ b^
SDH	7 (87.5)	
SDH and SAH	0 (0.0)	
EDH	1 (12.5)	
SAH	0 (0.0)	
Intracerebral hemorrhage	0 (0.0)	
SAH and Intracerebral hemorrhage	0 (0.0)	
ICH size		0.579^ b^
1mm, trace	3 (37.5)	
2mm	0 (0.0)	
3mm	1 (12.5)	
4mm	1 (12.5)	
5mm	3 (37.5)	
Midline shift presence		0.556^ b^
Yes	0 (0.0)	
No	13 (100)	
Skull fracture presence		0.528^ a^
Yes	11 (84.6)	
No	2 (15.4)	
Location of skull fracture		0.561^ b^
Parietal	8 (72.7)	
Temporal	0 (0.0)	
Facial	0 (0.0)	
Frontal	0 (0.0)	
Occipital	2 (18.2)	
Basilar	0 (0.0)	
Combined	1 (9.1)	
Type of skull fracture		<0.001^ b^
Linear	5 (45.5)	
Comminuted	2 (18.2)	
Depressed	2 (18.2)	
Diastatic	0 (0.0)	
Combined	2 (18.2)	
Displacement of skull fracture		0.334^ b^
Nondisplaced	11 (100)	
Minimally displaced	0 (0.0)	
Injury group		0.412^ a^
ICH only	2 (15.4)	
Skull fracture only	5 (38.5)	
Combined	6 (46.2)	

## Discussion

To date, there are only a handful of studies assessing outcomes in neurologically normal children presenting with mild TBI. Management of these patients varies significantly across institutions and providers. The National Institute for Health and Care Excellence (NICE) and the Scottish Intercollegiate Guidelines Network (SIGN) have both published guidelines recommending routine hospital admission for all adult patients with mild TBI who have documented injuries on initial brain imaging. On the other hand, the Brain Injury Guidelines (BIG) which are widely used by trauma centers in the United States advocate for the discharge of neurologically intact adult patients with minor ICH (BIG 1 risk group) from the emergency room after a period of observation. Our experience with this specific group of patients is that they tend to be admitted for observation but often have a seamless hospital course with a negligible rate of adverse outcomes. In this study, we sought to examine clinical outcomes for neurologically intact children with a traumatic minor ICH and/or simple skull fracture. We also attempted to identify patient-specific and radiological factors that are predictive of complications.

Mild TBI comprises a heterogeneous group of patients. Prior studies attempted to capture this group of patients using GCS scores, reported symptoms, neurological exams, and radiological findings on initial brain scans. Similar to what Greenberg et al. did [14], we elected to include patients with GCS ≥14 and documented injuries (ICH ≤5mm and/or simple skull fracture). However, contrary to prior studies that evaluated outcomes in patients with isolated ICH or ISF separately, we included both groups in our study and limited the ICH size to 5mm or less in an attempt to include a more representative sample of the lowest risk subgroup. We only included children aged three years or less since this age group tends to have the highest estimated annual rates of TBI-related emergency department visits [17]. 

The existing pediatric studies have reported various rates for neurosurgical interventions in this patient population, ranging from 6% to 43% [18-20]. Holsti et al. found that none of the patients with mild TBI and intracranial injuries required unplanned hospital admission [13]. On the other hand, Greenberg et al. evaluated pediatric patients with GCS ≥14, like our cohort, and found that 6.8% experienced clinically important neurologic decline and 5.1% underwent a neurosurgical intervention [14]. Burns et al. included all patients with traumatic ICH regardless of their initial GCS or ICH size and reported a 9.7% rate of critical care intervention [21].

Many of these studies failed to explain indications for given interventions, and rates of non-surgical complications like medical and neurological complications. We introduced SCS as a primary outcome. SCS was developed by combining the work done by the PECARN group in 2009, Washington et al. in 2012, and Greenberg et al. in 2014 [14-16]. SCS combines multiple clinical and radiological data points which makes it a more meaningful outcome for patients and their clinicians. In our relatively large cohort of 205 patients, the overall rates of clinical and radiologic deterioration were low. Only 6.3% of patients experienced SCS.

SCS was more common among male patients, patients where a report was filed with child protective services due to a concern for abuse/neglect, and patients with non-linear skull fractures (comminuted, depressed, and combined). The association between the remaining variables and SCS was not statistically significant but there seems to be a trend toward an increased rate of SCS in patients presenting with seizures, patients with ICH, patients with larger ICH (≥3mm), and patients with a combined injury (ICH and skull fracture). Nonetheless, a larger sample will be needed to determine if the trend we observed is significant. Healthcare services are generally costly, especially critical care. Based on some published estimates, the cost of a 24-hour ICU stay is $2,264 compared to $1,514 for the general ward in the United States [22]. Given how common closed head injuries are in childhood and the relatively short hospital stay, it would be highly cost-effective to efficiently triage these patients and provide appropriate disposition plans. Based on the results of our study, the common practice of admitting all children with mild TBI to the general ward or ICU may be unnecessary.

Our study attempted to add to the relatively scarce literature on the management of neurologically intact toddlers with traumatic closed-head injuries. To our knowledge, this is the largest cohort of its kind to date. Our findings confirm those of other studies, which is that clinical and radiological deterioration is rare in this patient population and especially in carefully selected low-risk subgroups. We recognize that this study has several limitations. Its retrospective nature introduces inherent biases and limitations. The data represents the management styles of a single tertiary center, which may not be fully generalizable. We collected our radiological data from the documented radiology reports in patients' charts without obtaining overreads to corroborate these findings. Another limitation of the study is the small sample size limiting its power. However, given the rarity of the outcome in question (SCS), meaningful conclusions can be made from our results.

## Conclusions

We, therefore, concluded that neurologically intact toddlers who are admitted with traumatic closed-head injuries rarely require medical, surgical, or critical care interventions. This study suggests that most children with asymptomatic mild TBI may not need ICU monitoring. This study also showed that a certain subset of patients might be at an increased risk of developing SCS. Future studies should focus on larger sample sizes and prospective analyses to obtain results that validate our findings and help address the necessary policy changes.
